# Mic13 Is Essential for Formation of Crista Junctions in Mammalian Cells

**DOI:** 10.1371/journal.pone.0160258

**Published:** 2016-08-01

**Authors:** Ruchika Anand, Valentina Strecker, Jennifer Urbach, Ilka Wittig, Andreas S. Reichert

**Affiliations:** 1 Institute of Biochemistry and Molecular Biology I, Heinrich Heine University, Medical Faculty, Universitätsstr. 1, 40225, Düsseldorf, Germany; 2 Functional Proteomics, SFB 815 Core Unit, Faculty of Medicine, Goethe-University, Frankfurt am Main, Germany; 3 Cluster of Excellence “Macromolecular Complexes”, Goethe University, Frankfurt am Main, Germany; 4 German Center of Cardiovascular Research (DZHK), Partner site RheinMain, Frankfurt, Germany; University of California Los Angeles, UNITED STATES

## Abstract

Mitochondrial cristae are connected to the inner boundary membrane via crista junctions which are implicated in the regulation of oxidative phosphorylation, apoptosis, and import of lipids and proteins. The MICOS complex determines formation of crista junctions. We performed complexome profiling and identified Mic13, also termed Qil1, as a subunit of the MICOS complex. We show that MIC13 is an inner membrane protein physically interacting with MIC60, a central subunit of the MICOS complex. Using the CRISPR/Cas method we generated the first cell line deleted for MIC13. These knockout cells show a complete loss of crista junctions demonstrating that MIC13 is strictly required for the formation of crista junctions. MIC13 is required for the assembly of MIC10, MIC26, and MIC27 into the MICOS complex. However, it is not needed for the formation of the MIC60/MIC19/MIC25 subcomplex suggesting that the latter is not sufficient for crista junction formation. MIC13 is also dispensable for assembly of respiratory chain complexes and for maintaining mitochondrial network morphology. Still, lack of MIC13 resulted in a moderate reduction of mitochondrial respiration. In summary, we show that MIC13 has a fundamental role in crista junction formation and that assembly of respiratory chain supercomplexes is independent of mitochondrial cristae shape.

## Introduction

Mitochondria are double-membrane enclosed organelles which are essential for a number of cellular processes such as energy conversion, apoptosis, calcium buffering, lipid trafficking and heme biosynthesis. The inner mitochondrial membrane is characterized by membrane protrusions into the matrix termed cristae. Mitochondria show a dynamic remodeling of cristae length, density, and shape depending on the cell type and/or the physiological and developmental stage [1]. Indeed, aberrant changes in mitochondrial cristae are associated with numerous human diseases including Alzheimer’s disease, Parkinson’s disease, Wilson disease, and hereditary mitochondrial hypertrophic cardiomyopathy. It is not understood whether mitochondrial cristae alteration is a cause or consequence of these diseases. Cristae divide the inner membrane (IM) into the cristae membrane (CM) and the inner boundary membrane (IBM) which runs parallel to the outer membrane. Cristae are physically connected to the IBM via crista junctions (CJs)—highly curved pore- or slit-like membrane structures with a diameter ranging from 12 to 40 nm [2–4]. CJs are proposed to play an important role in cristae remodeling during apoptosis and to act as a diffusion barrier between IBM and CM [1, 2, 5]. The IBM is rich in the proteins required for fusion/fission, protein import or signaling whereas the CM predominately contains proteins required for oxidative phosphorylation [6, 7]. This uneven, yet dynamic, distribution of various mitochondrial proteins between IBM and CM is likely mediated via CJs [1, 2, 5]. The presence of CJs also creates distinct aqueous compartments: the inter-membrane space between IBM the OM and the intracristal space. The diameter of CJs is proposed to be remodeled for example during apoptosis when cytochrome *c* is released from the intracristal space [8]. Also various metabolites such as protons, ADP and other apoptosis effectors reside in the intracristal space. Therefore, the shape and size of CJs was proposed to determine rates of ATP production and thus may be fundamental for regulation of bioenergetics [5, 9].

We have previously identified and characterized MIC60/Fcj1 in yeast cells as the first protein required for crista junction formation which was localized to CJs by immunoelectron microscopy [10]. Cells lacking MIC60/Fcj1 in baker’s yeast have no CJs showing concentric stacks of membrane vesicles within the matrix. Independent studies have later identified a large heterooligomeric complex containing MIC60/Fcj1 as a core constituent playing a role to maintain cristae structure [11–13]. Following a uniform nomenclature, the complex is named as MICOS, “mitochondrial contact site and cristae organizing system” and its protein subunits MIC10 to MIC60 [14]. Thus, till date six subunits MIC60/Fcj1, MIC12/Aim5, MIC19/Aim13, MIC27/Aim37, MIC10/Mio10, and MIC26/Mio27, are reported in yeast. The MICOS complex is highly conserved from yeast to humans with the majority of the proteins also having mammalian homologs [15–17]

MIC60/Mitofilin is the mammalian homolog of MIC60/Fcj1. Apart from MIC60, the mammalian MICOS complex contains at least five other components, MIC10/Minos1, MIC19/CHCHD3, MIC25/CHCHD6, MIC26/APOO, and MIC27/APOOL [15–17]. CHCHD10 causative for frontotemporal dementia-amyotrophic lateral sclerosis was recently added to the growing list of subunits of MICOS [18]. The depletion of any of these subunits of the MICOS complex has been shown to alter cristae morphology. Reduced levels of MICOS components have deleterious effects on various cellular processes. For example, loss of MIC60/Mitofilin causes decreased cellular proliferation and increased sensitivity to induction of apoptosis [19]. Apparently, these cells are more prone to apoptosis due to the accelerated release of cytochrome *c* exemplifying the importance of CJs in regulating apoptosis [20]. MIC60/Mitofilin interacts with a variety of proteins such as MIC19/CHCHD3, DISC1, SAM50 linking the MICOS complex to cellular processes such as mitochondrial protein import and modulation of neuronal activity [11, 12, 17, 21]. Loss of MIC19/CHCHD3 leads to reduced cell proliferation and increased autophagy [21]. Depletion of MIC25/CHCHD6 causes alteration in cristae morphology and reduced cell growth, ATP production and oxygen consumption [22–24]. Moreover, altered levels or post-translational modifications of MICOS subunits are observed in a set of diverse human diseases such as epilepsy, Down syndrome, Parkinson’s disease, diabetes, cardiomyopathy [17]. Overexpression of MIC60/Mitofilin in a transgenic mouse model protects against cardiac dysfunction normally observed after drug-induced diabetes mellitus indicating a protective role of MICOS in diabetes [25]. Altered levels of MIC19/CHCHD3 are found in disease models for familial amyotrophic lateral sclerosis and ischemia [26]. Downregulation and overexpression of MIC25/CHCHD6 altered chemosensitivity of cancer cells to genotoxic anticancer drugs indicating its potential as a possible target for cancer therapeutics [22]. Overall, the physiological and pathophysiological importance of the MICOS complex is evident, however, the molecular understanding and the involved mechanisms are still unclear.

Recently, we have identified two apolipoproteins, MIC26/APOO and MIC27/APOOL, as subunits of the MICOS using a complexome profiling approach. [16, 27, 28]. Classically, apolipoproteins bind lipids in order to transport them within the lympathic and circulatory system. Recently, we have deciphered the role of MIC26/APOO and MIC27/APOOL in maintaining cristae morphology. Downregulation of MIC27/APOOL resulted in reduced numbers of cristae and appearance of small concentric structures which are partly branched and interconnected. Recombinant MIC27/APOOL can bind cardiolipin *in vitro* indicating that function of MIC27/APOOL is linked to its ability to bind the mitochondrial lipid cardiolipin. MIC26/APOO was previously known as a secreted glycosylated lipoprotein. MIC26/APOO levels are increased in diabetic heart tissue and in blood plasma of patients suffering from acute coronary syndrome [29–31]. We found that apart from the glycosylated form, MIC26 is also present in a non-glycosylated form which is localized to the inner mitochondrial membrane [27]. It physically interacts with components of the MICOS complex. Downregulation and overexpression of MIC26/APOO causes aberrant cristae morphology. Interestingly, both MIC26/APOO and MIC27/APOOL regulate each other’s levels antagonistically and they positively regulate levels of MIC10 and tafazzin, an enzyme required for cardiolipin remodeling in mitochondria [27]. The binding of MIC27/APOOL to cardiolipin and the observed changes in tafazzin levels upon downregulation of MIC26/APOO and MIC27/APOOL point to a role of the MICOS complex in lipid homeostasis in mitochondria.

Here we applied ‘complexome profiling’ to identify novel MICOS components [32]. Apart from MIC26/APOO and MIC27/APOOL which we characterized previously, we have identified MIC13 as a novel MICOS component using this approach consistent with a recent study [33]. Here, we generated knockouts of MIC13 using CRISPR/Cas method and characterize the role of MIC13 in formation of crista junctions.

## Results

### Complexome profiling identifies MIC13 as a novel MICOS subunit

**Fig 1:**
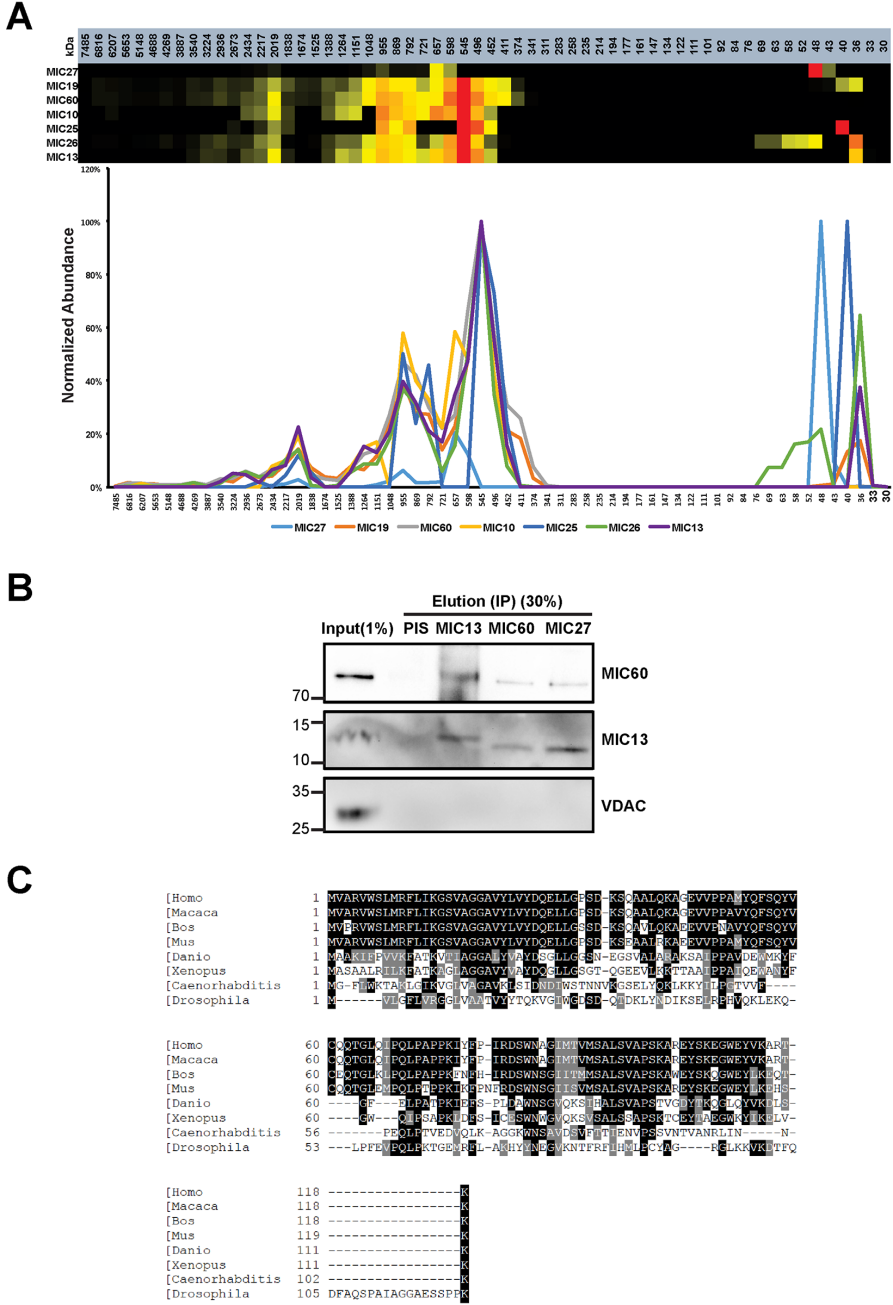
MIC13 is identified as MICOS subunit using complexome profiling. (A) The graph shows the normalized occurrence of the proteins which cocluster with the MIC60 and other MICOS components in control HEK293 cells. Using this complexome profile, MIC13 is identified as MICOS component. (B) Endogenous MIC13, MIC60 and MIC27 antibodies were used for coimmunoprecipitation. Preimmuneserum (PIS) was used as the control. Endogenous MIC13 could pull down MIC60 and reciprocally in control 143B cells, MIC60 and MIC27 can pull down MIC13, indicating that MIC13 is the part of the MICOS complex. (C) Sequence alignment of MIC13 shows that it is conserved from *Caenorhabditis elegans* to higher eukaryotes.

In order to identify novel subunits of the MICOS complex in mammalian systems we applied a proteomics method termed ‘complexome profiling’ [32]. Using this approach with bovine mitochondria we identified several mitochondrial complexes and their constituents including the MICOS complex and its known subunits MIC60/Mitofilin, MIC19/CHCHD3, and MIC10/MINOS1 [28]. Subsequently, we have identified and characterized MIC26/APOO and MIC27/APOOL as novel MICOS subunits [27, 28, 34]. Here we followed a similar strategy and solubilized mitochondria isolated from HEK293 cells using digitonin, separated native macromolecular protein complexes by large-pore blue native gel electrophoresis, and divided the gel lane in 60 equal gel slices. Quantitative mass spectrometry was performed for all gel slices and each identified protein was represented according to its relative abundance at the corresponding size. Hierarchical clustering was used to identify protein clusters with similar distribution profiles. We found that MIC13 also clustered with MICOS subunits in this complexome analysis compatible with the idea that MIC13 is a novel subunit of the MICOS complex (Fig 1A). We verified the interaction of MIC13 with MIC60 and MIC27 using coimmunoprecipitation experiments (Fig 1B). Human MIC13 is a small protein of 118 amino acids which is conserved from *C*. *elegan*s to human (Fig 1C). We did not find any apparent homolog of MIC13 in *Saccharomyces cerevisiae*. Overall, we conclude that MIC13 is a novel *bona fide* subunit of the MICOS complex consistent with a recent study [33].

### MIC13 localized to mitochondrial inner membrane

**Fig 2:**
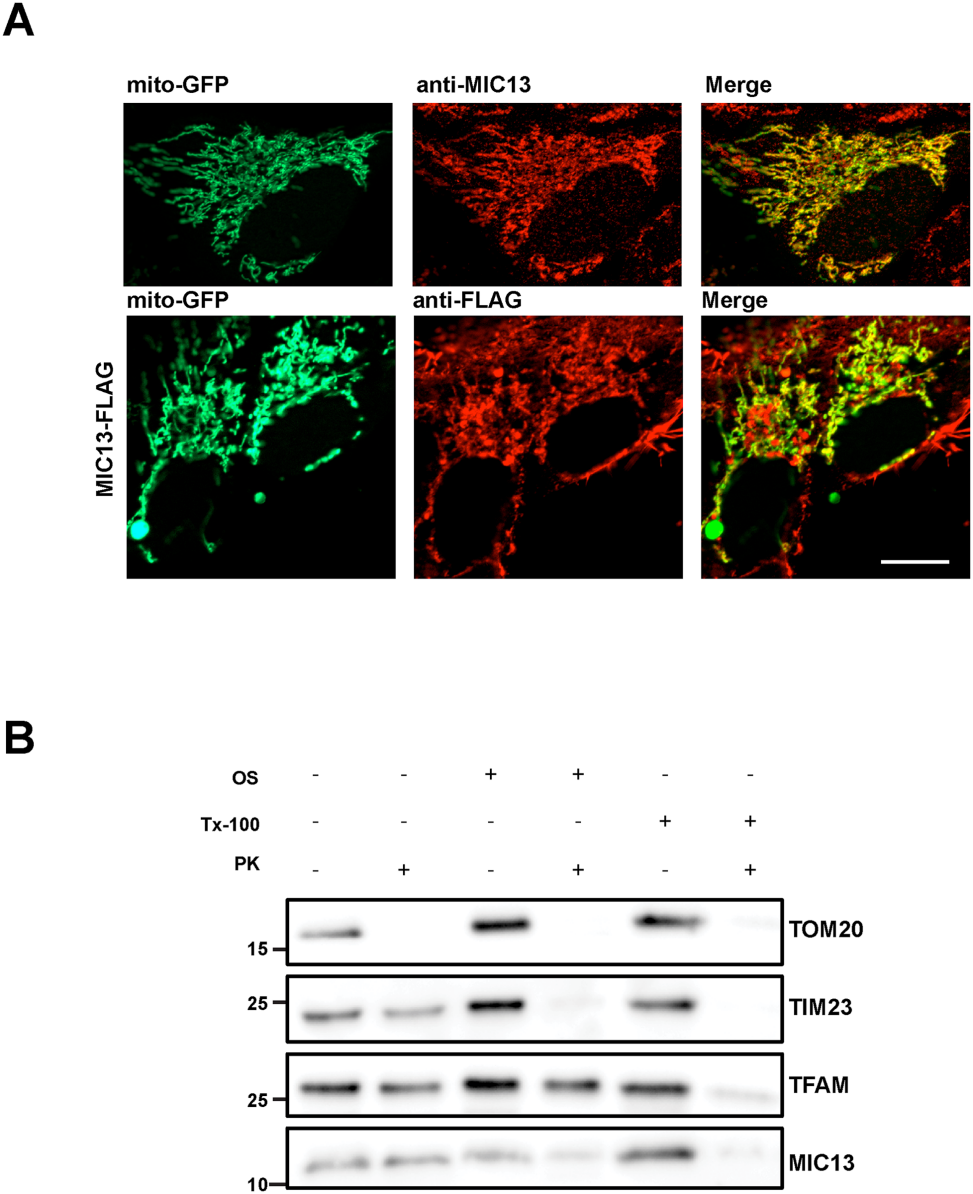
MIC13 localizes to inner mitochondrial membrane. (A) Representative images of mitochondria (marked by mito-GFP, green) and endogenous MIC13 (using MIC13 antibody, red) or MIC13-FLAG (marked by anti-FLAG) in control 143B cells. Merge shows the colocalization of mitochondria and MIC13. Scale bar 10μm. (B) Isolated mitochondria of control 143B cells were swelled by osmotic shock (OS) and then treated or untreated with Proteinase K (PK). Triton-x-100 (Tx100) was used to permeabilize all the membranes. TOM20, TIM23 and TFAM were used for outer membrane, inner membrane and matrix marker respectively.

We determined the subcellular localization of endogenous MIC13 using immunofluorescence microscopy. For that we immunostained human 143B cells using a MIC13-specific antibody. Mitochondria were visualized using a mitochondrially targeted GFP. We clearly observed a colocalization of MIC13 with mitochondrial structures demonstrating the mitochondrial localization of endogenous MIC13 (Fig 2A). MIC13 is uniformly distributed along the whole length of mitochondria. The antibody used was validated by western blot analysis showing a protein band at the expected size of approximately 10 kDa. Furthermore, we expressed MIC13 harboring a C-terminal FLAG-tag (MIC13-FLAG) in 143B cells. These cells were immunostained using an anti-FLAG antibody. The majority of MIC13-FLAG was present on mitochondria confirming the mitochondrial localization of MIC13 (Fig 2A).

We next investigated the submitochondrial localization of MIC13 using a standard protease protection assay. We found that MIC13 was susceptible to degradation after addition of proteinase K in mitoplasts behaving like the inner membrane protein TIM23 (Fig 2B, lanes 3 and 4). Outer membrane protein, TOM20 is degraded by proteinase K in the intact mitochondria (Fig 2B, lanes 1 and 2) The matrix protein TFAM is resistant to proteinase K treatment in mitochondria (Fig 2B, lanes 1 and 2) and in mitoplasts (Fig 2B, lanes 3 and 4) demonstrating that matrix proteins are protected from degradation by proteinase K. All proteins were degraded by Proteinase K after complete solubilization of membranes by Triton-X-100 (Fig 2B, lanes 5 and 6). Overall we conclude that MIC13 is an inner membrane protein consistent with its role as a novel subunit of the MICOS complex known to localize to the inner membrane.

### MIC13 is essential for the formation of mitochondrial crista junctions

**Fig 3:**
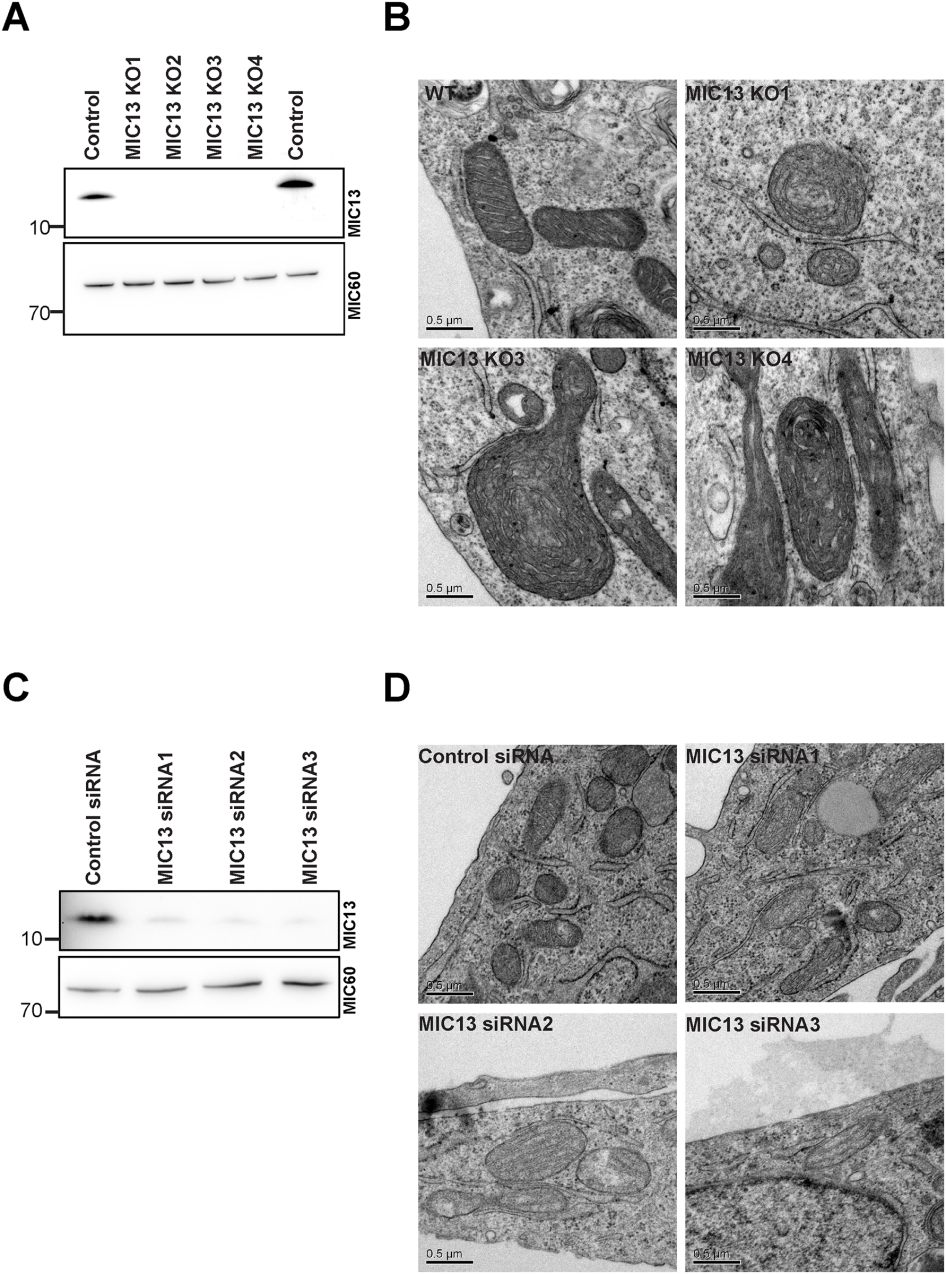
MIC13 KO cells have no crista junctions. (A) Immunoblot of MIC13 showing the complete loss of the protein in the knockout cell lines. (B) Representative EM images of control and MIC13 KO mitochondria (we analyzed approx. 40 to 50 mitochondria of each MIC13 KO cell line (N = 2). There is a complete loss of CJs in MIC13 KO cells whereas in control cells 1 to 5 CJs were observed in nearly all sections (40 to 50 mitochondria, N = 2). (C) Immunoblot of MIC13 in HeLa cells where MIC13 is depleted using SiRNA. (D) Representative EM images of control and MIC13 siRNA mitochondria.

We generated MIC 13 knockout cells (MIC13 KO cells) using the CRISPR/Cas system. The double nickase Cas9 enzyme was targeted to specific sites of exon 2 of MIC13 to create deletions or insertions eventually resulting in cells lacking a functional MIC13 protein. The use of double nickase strategy drastically reduces the chances of non-specific targeting [35]. We first screened single clonal populations of cells that lack any visible immunoreactivity for the MIC13-specific antibody. We obtained several clonal cell populations lacking MIC13 which was validated in four cell lines by western blot analysis (Fig 3A). These are the first cell lines where MIC13 is knocked out serving as a valuable tool to study the function of MIC13 and the MICOS complex. These cell lines were viable and could be cultured in normal MEM media supplemented with bovine serum indicating that MIC13 is not essential for the viability of these cells. Apart from these MIC13 KO cells, we also depleted MIC13 in the HeLa cells using siRNA to obtain results using an independent approach. These cells also show considerably low levels of endogenous MIC13 protein (Fig 3C). With both cell systems we investigated the role of MIC13 in regulating mitochondrial cristae organization by electron microscopy. We observed complete loss of crista junctions in all the clones of MIC13 KO cells as in a total of 135 mitochondrial sections observed in three MIC13 KO cell lines no crista junction was observed (Fig 3B). The cristae were arranged in an onion-like fashion and lacked any visible connection to inner boundary membrane (IBM). HeLa cells depleted of MIC13 also showed a similar phenotype, yet occasionally crista junctions remained visible which we attribute to incomplete depletion of MIC13 (Fig 3D). Quantitative analysis of areas of mitochondrial sections in electron micrographs indicate that mitochondria in MIC13 KO cells are on average larger in size and show a larger range of observed values compared to wild type cells (S1A Fig). This increase in mitochondrial area is significant for two MIC13 KO cell lines whereas a third cell line only showed a slight tendency in the same direction which, however, was not significant. Given the fact that mitochondria appeared packed with onion-like stacks of cristae we decided to analyze whether the area of cristae is higher in MIC13 KO cells. For this we chose one of the KO cell line showing higher increase in mitochondrial area (MIC13 KO3). Our analysis revealed that area of cristae were markedly increased in the MIC13 KO3 cell line. This increase was not caused by swelling but by elongation of cristae stacks. This is compatible with the idea that an increase in mitochondrial size is caused by enlarged cristae stacks in KO cells (S1B Fig). Taken together, our data demonstrate that MIC13 is essential for crista junction formation in mammalian cells and determines mitochondrial size and ultrastructure. This is in accordance with MIC13 being an important subunit of MICOS complex.

### MIC13 is required for efficient assembly of the MICOS complex

**Fig 4:**
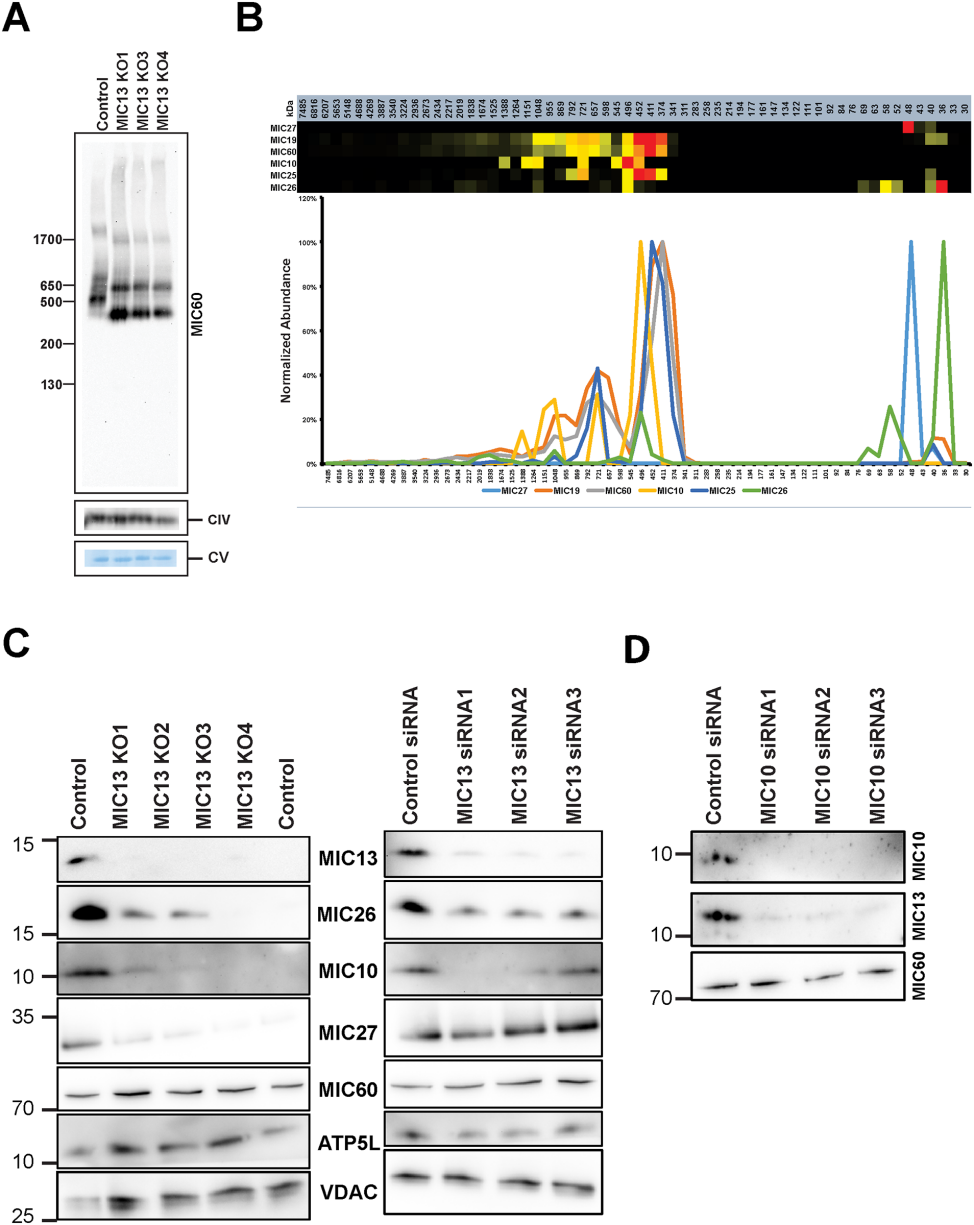
MIC13 knockout results in smaller but assembled MICOS complexes. (A) Protein complexes were isolated by blue-native electrophoresis (BNE) on 3 to 18% acrylamide gradient gels immuno-decorated against MIC60, subunit coxVIa/b of complex IV and ATP synthase was shown on blot to demonstrate equal loading of samples and no effect. In control HEK293 cells, MICOS is detected around 500 KDa. Deletion of MIC13 leads to smaller MICOS complex. (B) Complexome profiling of the MICOS complex in MIC13 KO cells demonstrate the smaller MICOS complex (subcomplex) comprises of MIC60, MIC19 and MIC25. (C) Immnuoblot showing the steady state levels of various MICOS components in MIC13 KO cells as well as cells treated with MIC13 siRNA. There is reduction of MIC10, MIC27 and MIC26 upon deletion of MIC13. (D) Immunoblot from the cells depleted of MIC10 and probed for MIC13 and MIC60.

**Fig 5:**
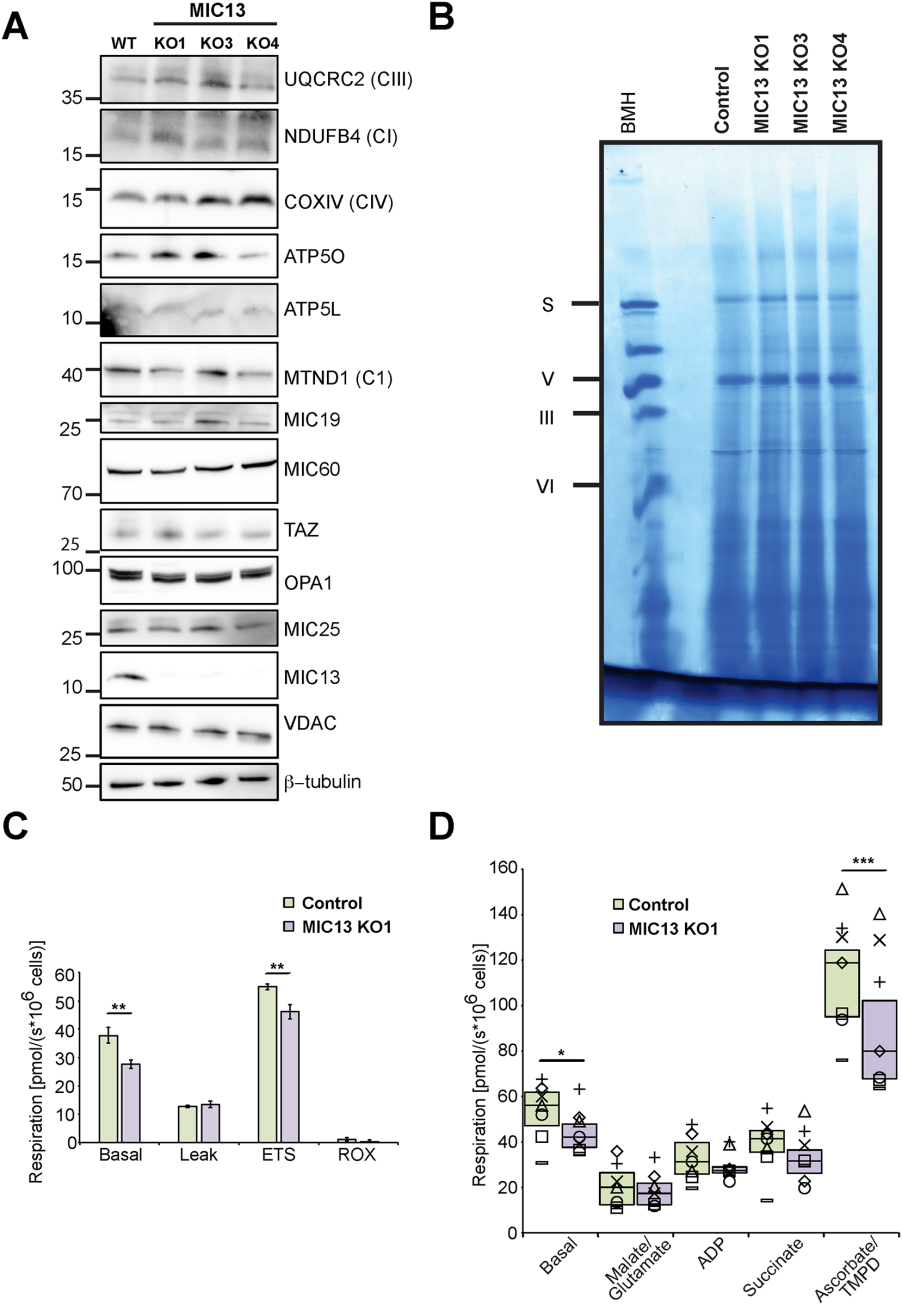
Stability and assembly of respiratory chain complexes are unchanged upon MIC13 depletion. (A) Immunoblot showing the steady state levels of various subunits of RC. (B) Blue-native electrophoresis of protein complexes stained with coomassie to detect major respiratory chain complexes in control and MIC13 KO cells. (C) Oxygen consumption rates of control and MIC13 KO1 cells are plotted as histogram (Mean±SE, N = 6). The basal, leak, ETS (electron transport system capacity or maximum respiration) and ROX (residual respiration) are shown for control and MIC13 KO1 cells. **, p value < 0.01. (D) Oxygen consumption rates measured in digitonin permeabilized cells after addition of indicated respiratory substrates and inhibitors. Basal, malate/glutamate (Complex I), ADP, succinate (Complex II/III), and ascorbate/TMPD (Complex IV) respiration are represented as box plots on which the median and 75/25 percentiles is plotted. In addition, all data values from seven individual experiments are plotted (Δ = Exp.1, × = Exp. 2, ◊ = Exp. 3, – = Exp. 4, □ = Exp. 5, ○ = Exp. 6, + = Exp. 7). *, p value < 0.05; ***, p value < 0.001.

Next we aimed to check whether MIC13 is required for the stability and/or assembly of the MICOS complex. For this purpose, we studied MICOS complex using blue native electrophoresis in MIC13 KO cells and corresponding controls. In control cells the MICOS complex migrated at molecular weights of approximately 550 kDa, 950 kDa, and 2000 kDa (Figs 1A and 4A). In the absence of MIC13, the MICOS (sub)complex consistently showed a reduced molecular weight with values of approximately 400 kDa, 680 kDa, and 1800 kDa (Fig 4A and 4B). In order to study the composition of MICOS complex in the absence of MIC13 we subjected gel slices obtained from a blue native gel for quantitative mass spectrometry and complexome profiling. As expected from the blue native data, we observed a clear shift in the peak MICOS complex in MIC13 knockout cells (compare Figs 1A and 4B). The complexome profiles reveal that after deletion of MIC13 the MICOS complex predominantly consists of only MIC60, MIC19 and MIC25 while MIC26 and MIC27 appear as low molecular weight complexes (Fig 4B). Also MIC10 appears to form now separate complexes with approximate sizes of 1100 kDa, 720 kDa, and 500 kDa indicating that loss of MIC13 destabilizes the MICOS complex. We further investigated the steady state levels of other MICOS components in MIC13 KO cells and in MIC13 depleted cells. In both cell types we observed strongly reduced levels of MIC10, MIC26 and MIC27 compared to control cells, however, MIC60 levels remained unchanged (Fig 4C). MIC27 stability differs between the KO cells and transient siRNA, suggesting the differential effect on long term stability of some of the MICOS components. Steady state levels of MIC25 and MIC19 are unchanged upon MIC13 depletion (Fig 5A). Based on these observations we suggest that MIC13 is required for assembly of MIC10, 26 and 27 with the remaining subunits of the MICOS complex. However, the assembly of MIC60, MIC19 and MIC25 in the MICOS subcomplex is apparently independent of MIC13. We speculate hierarchical steps in the formation of MICOS complex wherein MIC60, MIC25 and MIC19 first form an intermediate complex and then bind to another intermediate subcomplex consisting of MIC13, MIC10, MIC26, and MIC27. If this is true we hypothesize that depletion of any of its constituents could make this subcomplex unstable and leads to subsequent degradation of its remaining constituents. This could explain the significant loss of MIC26, MIC27 and MIC10 in MIC13 KO cells. In order to verify this we decided to perform a reciprocal experiment and depleted MIC10 in HeLa cells using siRNA and probed for MIC13 protein levels. In line with our hypothesis, we observed a clear reduction of MIC13 in these cells (Fig 4D) indicating that MIC13 and MIC10 are reciprocally stabilized as they are the part of same MICOS subcomplex.

### MIC13 is dispensable for OXPHOS complex formation but moderately affects respiration

It was proposed recently that cristae morphology is critical for proper assembly of respiratory chain supercomplexes [36]. Using the MIC13 KO cells we thus aimed to evaluate the role of crista junctions and cristae morphology on maintaining the structure and stability of OXPHOS complexes in mitochondria including respiratory chain supercomplexes. We initially examined the steady state levels of several subunits of complexes of the respiratory chain but we did not find any obvious and consistent differences between control and MIC13 KO cells (Fig 5A). To investigate this we analyzed respiratory chain complexes using BN-PAGE and subsequent complexome profiling in control and MIC13KO cells. Standard coomassie stain of major OXPHOS complexes in control and MIC13 Knockout cells does not reveal any obvious difference in the stability of any of the respiratory chain complexes (Fig 5B). We also compared the compositions of subunits of various OXPHOS complexes in control and MIC13 KO cells using complexome profiling. We did not find any major change in composition of any of the respiratory chain complex between control and MIC13 KO cells (S2 Fig). We further investigated the role of MIC13 in mitochondrial respiration. We observed a decrease in basal and maximal respiration of MIC13 KO cells compared to control cells (Fig 5C). We performed substrate inhibitor titration protocol to examine whether the activity of a specific respiratory complex is compromised in MIC13 KO cells. Consistent with our basal respiration experiment, we observed a significant decrease in basal respiration of MIC13 KO compared to control (Fig 5C and 5D). We observed only a slight decrease in Complex I (glutamate and malate) and Complex II/III (succinate) driven respiration of MIC13KO which was not statistically different compared to control. However, Complex IV (ascorbate/TMPD) driven respiration was significantly reduced in MIC13 KO (Fig 5D). From these results, we conclude that crista junctions are not required for the assembly and stability of major respiratory chain complexes and that basal respiration is only reduced moderately in the absence of crista junctions. This reduced basal respiration could be due to a combined modest reduction in the activity of all respiratory complexes. Overall we demonstrate a minor but significant influence of CJs on mitochondrial respiration.

We also checked mitochondrial morphology of MIC13 KO cells to observe any influence of crista junctions on mitochondrial tubulation. We stained mitochondria using cytochrome c in control and MIC13 KO cells (S3 Fig). We observed tubular mitochondria in both control and MIC13 KO cells, indicating that crista junctions are dispensable for mitochondrial tubular morphology.

## Discussion

Mitochondrial crista junctions are crucial for mitochondrial structure and function. Using complexome profiling approach, we identified MIC13 as a novel subunit of MICOS complex. MIC13 was also reported as a subunit of MICOS complex in a recent publication [33]. Our data confirms the presence of MIC13 in MICOS complex. We have generated the first knock out cell line of MIC13 using the CRISPR/Cas method. The knockout cell approach eliminates possible artefacts due to insufficient depletion of the protein of interest e.g. by a siRNA approach. MIC13 KO cells showed complete loss of crista junctions, hence proving an essential requirement of MIC13 in the formation of crista junctions. The cristae structure in MIC13 KO cells resembles the one that is observed upon deletion of certain MICOS subunits in yeast cells and mammalian cells with cristae arranged as an onion slices and devoid of any connection with IBM. We also observed a slight increase in area of mitochondria which correlates with the increased average area of cristae in the MIC13 KO cells suggesting the influence of CJs in determining the number and size of cristae per mitochondria. This strongly resembles the situation in Δ*MIC60/FCJ1* cells in baker’s yeast [10]. Knockout cells of MIC25, which were generated using TALEN approach, did not show such drastic cristae phenotypes [23]. Contrary to earlier observations including ours [10, 19] knockdown cells of MIC60 showed circular cristae [23]. These differences in cristae phenotype of various subunits of MICOS indicate that they have different roles in the formation and maintenance of CJs. In this regard, the study of true knockout cell models might promote our understanding of the mechanisms mediating formation of cristae and crista junctions.

We observed that upon deletion of MIC13, the MICOS complex remains intact but is reduced in size. This smaller complex still contains the MIC60-MIC19-MIC25 subcomplex. MIC60 is the major component of MICOS complex. Despite the normal levels of MIC60 (and MIC60-MIC19-MIC25 subcomplex), crista junctions are completely absent in MIC13 KO cells implying that intact MIC60-MIC19-MIC25 subcomplex is not sufficient to maintain crista junctions. These results clearly show the importance of MIC13-MIC26-MIC27-MIC10 subcomplex for crista junction maintenance. Our study supports the hierarchical assembly of the MICOS complex which was also suggested earlier [37, 38]. It was suggested previously that MIC13 is required for the stability of MIC10 [33]. In a reciprocal experiment, we observed that MIC13 and MIC10 regulate each other´s stability. It is not understood that how this regulation is carried out. One possibility could be that during normal MICOS complex assembly MIC13 and MIC10 part of a transient MIC10-MIC13-MIC26-MIC27 subcomplex and depletion of one of the subunits of this subcomplex leads to degradation of the remaining subunits. Thus, we cannot conclude whether the loss of crista junctions is a direct or indirect consequence of MIC13 deletion. The crista junctions’ phenotype in MIC13 KO cells could also be explained by a lack of MIC10. Mic10 forms large oligomers which are required for the formation of CJs [39, 40].

It was proposed that aberrant cristae structure could influence the assembly of the respiratory chain complexes [36]. We cannot support this as in our study loss of CJs in MIC13 KO cells does not influence assembly of major respiratory chain complexes. This is in accordance to the yeast phenotypes when Δ*mic60* and Δ*micos* cells were analyzed [41]. However, despite the normal assembly of the respiratory complexes, basal respiration was moderately reduced in the MIC13 KO cells. This indicates a functional role of crista junctions for proper functioning of respiratory chain complexes. Furthermore, we did not detect a role of CJs for allowing maintenance of tubular mitochondria. Overall, complete loss of CJs in the mammalian cells is apparently dispensable for viability of cells, OXPHOS complex assembly and mitochondrial morphology but affects basal respiration.

Using profile-profile comparison tools, a recent study indicated that Mic12/Aim5 from baker’s yeast could be an ortholog of MIC13 [37]. However, the sequence similarity between these genes is very low and was not detected by us or a recent evolutionary analysis of the MICOS complex [38]. Mic12 was recently shown to be required for the coupling of two MICOS subcomplexes in baker’s yeast [42] which support the idea that MIC13 and MIC12 have similar functions. Still, future studies are needed to test whether human MIC13 is a true orthologue of Mic12.

## Material and Methods

### Cell lines and cell culture

All the cells were cultured using Minimal Essential Media (M4655, sigma) with 1g/L of glucose supplemented with 10% Fetal Bovine Serum, 1mM Sodium Pyruvate (Gibco) and 1% Penicillin and streptomycin (Gibco), incubated at 37°C at 5% CO2 incubator. HEK (Flp-InTM T-RExTM 293) cells were obtained from Björn Stork from Institute of Molecular Medicine I, Düsseldorf, Germany.

### Generation of MIC13KO cells using CRISPR/Cas method

For generation of knockouts of the MIC13, we selected the primers for targeting the Exon2 of MIC13 gene. We used website crispr.mit.edu for generation of primers using double nickase platform. We used these two sets of primers which have higher scores using this website. Pair 1: bottom strand GCAGCTCCTGGTCGTACACCAGG and top strand GAGCCAGGCAGCCCTACAGAAGG. Pair 2: bottom strand CTGGTCGTACACCAGGTAGACGG and top strand AGGCAGCCCTACAGAAGGCTGGG. Primers were generated and annealed to have overhangs of Bst1 restriction site, which were integrated in px335 (addgene) vector at Bst1 (NEB) site in its cloning site. The respective pair of plasmids for targeting the top and bottom strands respectively were transfected in HEK293 (Flp-InTM T-RExTM 293) cells using Effectene reagent (Qiagen) (transfection was done according to manufacturer’s protocol). After two days of transfection, cells were trypsinized and sorted as single cells in a 96 well plates using FACS. These single cells were allowed to grow for 2–3 weeks until a visible colony could be found in the well. The single colony was trypsinized and further cultured. The cell lysate from these colonies was screened using western blotting for ones having no immunoreactivity to MIC13 antibody. The cells with no MIC13 protein were termed knockouts and used for further studies.

### Transfections siRNA

HeLa cells were plated on a petri dish overnight for transfection of the siRNA of MIC13 and MIC10 (Invitrogen, shealth siRNA). Lipofectamine RNAiMax (Invitrogen) was used for the transfection of the siRNA using the manufacturer’s protocol. We used 20nM final concentration for each of the siRNA. Scrambled siRNA provided by the manufacturers was used as control. The sample were collected after 72 h of transfection and prepared for western blotting or electron microscopy.

### Electron microscopy

The cells were cultured on the petri dish with 80% confluency. The cells were chemically fixed using 3% glutaraldehyde buffered with 0.1M sodium cacodylate buffer, pH 7.2 on the petri dish. After fixation, cells were scraped using a cell scraper and collected as a pellet in a mini tube. The cell pellet is washed with 0.1M sodium cacodylate, pH 7.2 and subsequently embedded in 2% agarose. The staining was performed using 1% osmium tetroxide for 50min followed by 1% uranyl acetate/ 1% phosphotungstic acid for 1h. These samples were dehydrated using the graded acetone series and specimens were embedded in spur epoxy resin for polymerization at 65°C for 24 h. The ultrathin sections were prepared using the microtome. Imaging was performed using Transmission electron microscope (Hitachi, H600) at 75V. Image were acquired using Bioscan model 792 (Gatan). Images were visualized and analyzed using ImageJ software. For measuring the size parameter of individual mitochondria, ImageJ Analyze Plugin was used.

### Mitochondrial isolation and submitochondrial localization

The cells were scraped from the petri dish using a cell scarper and pelleted at 500 g for 5 min. This pellet was resuspended in an isotonic buffer (220 mM mannitol, 70 mM sucrose; 1 mM EDTA and 20 mM HEPES, pH 7.5 and 1x protease inhibitor cocktail (Roche)) for 5mins. Cells were lysed by mechanical rupture by repeatedly passing through a syringe needle of 20G cannula for 20 times. The suspension was centrifuged at 1000 g for 5 min to remove the cell debris and nuclei. The supernatant which contains heavy membrane was further centrifuged at 8000 g for 10 min to pellet crude mitochondria. This crude mitochondria were used for submitochondrial localization of MIC13 in mitochondria, where the swelling of the mitochondria was performed using 10mM HEPES, pH 7.5 for 10mins on ice. 1% TritonX-100 was used to solubilize all the membranes. Proteinase K was used at the final concentration of 50μg/ml for 10mins. The reaction was stopped using 2mM PMSF.

### SDS electrophoresis and western blotting

The sample for the western blotting was prepared by scraping the cells in PBS. The protein extraction was done using RIPA lysis buffer. The amount of protein was determined using Bradford reagent and spectrophotometer for equal loading of a gel. The samples were prepared in Laemmli loading buffer. 10–20% precast Tricine gel (Invitrogen) was used (particularly for MIC13 as it is a small protein). The proteins were transferred onto nitrocellulose membrane and probed for various antibodies. Anti-HRP secondary antibodies were used. We used following antibodies for immunoblotting, MIC13 (Pineda, Berlin, polyclonal antibody raised in rabbit using following peptide CKAREYSKEGWEYVKARTK), MIC27/APOOL (Atlas Antibodies, HPA000612), MIC26/APOO (Thermo-Fisher, MA5-15493) MIC60/Mitofilin (Pineda, Berlin, polyclonal antibody raised in rabbit against human IMMT using the peptide CTDHPEIGEGKPTPALSEEAS), MIC10/MINOS1 (Pineda, Berlin, polyclonal antibody raised in rabbits against CQHDFQAPYLLHGKYVK), MIC25/CHCHD6 (cell signaling), VDAC (abcam), β-tubulin (Cell signaling), MIC19/CHCHD3 (abcam), ATP5L (proteintech), ATP5O (abcam), COXIV (abcam), NDUFB4 (abcam), UQCRC2 (abcam), MTND1 (abcam), TOM20 (Proteintech), TIM23 (BD biosciences), human TAZ1 (gift from Steve Claypool). Recording and visualization of chemiluminescent signals were done using VILBER LOURMAT Fusion SL (Peqlab).

### Fluorescence microscopy

For colocalization of MIC13 with mitochondria, mitoGFP targeting into mitochondrial matrix was transfected into 143B cells using Effectene transfection reagent (protocol of manufacture was followed). For overexpression of MIC13, pxFLAG-MIC13 was cotransfected with mitoGFP. After 24h of transfection the cells were fixed with 4% paraformaldehyde, permeabilized with 0.15% Triton-X100 and blocked using 3%BSA. The anti-MIC13 or anti-FLAG were used respectively to visualize endogenous or over expressed MIC13. Imaging was done using Zeiss Apotome microscope. For mitochondrial morphology analysis, control and MIC13 KO cells were fixed and stained for Alexa-488 tagged cytochrome c antibody (BD bioscience). Images were acquired using Zeiss LSM 710. Images were analyzed and prepared using Zeiss software using smooth filter.

### Respirometry measurements

Respirometry experiments were performed with an Oxygraph-2k (Oroboros). To study the oxygen flux, oxygen consumption was observed according to Pesta and Gnaiger [43]. Basal respiration was measured in intact cells in complete growth media. The ‘Leak’ state was induced by addition of oligomycin (2 μg/ml) (Sigma). For maximal respiration (the electron transport system capacity (ETS)), CCCP (Sigma) was added in steps of 0.25 μM to a maximum final concentration of 4.5 μM. The residual oxygen consumption (ROX) was observed after addition of rotenone (0.5 μM) (Sigma) and antimycin A (2.5 μM) (Sigma).

To study respiratory chain complexes a protocol from Kuznetsov *et al*. [44] was used. Before the oxygen consumption measurements, the cells were pelleted and resuspended in MIR05 buffer (0.5 mM EGTA, 3 mM MgCl_2_, 60 mM lactobionic acid, 20 mM taurine, 10 mM KH_2_PO_4_, 20 mM HEPES, 110 mM D-Sucrose, 1 g/l BSA, fatty acid free). Cells were permeabilized by addition of 10 μg digitonin (Sigma) per million cells in a total volume of 2 ml. Different substrates and inhibitors were consecutively added as follows: 10 mM glutamate (Sigma), 5 mM malate (Sigma), 5 mM ADP (Sigma), 0.5 μM rotenone (Sigma), 10 mM succinate (Sigma), 5 μM antimycin A (Sigma), 0.5 mM *N*,*N*,*N′*,*N′*-Tetramethyl-*p*-phenylenediamine dihydrochloride (TMPD) (Sigma), 2 mM ascorbate (Sigma) and 10 μM cytochrome C (Sigma).

### Isolation of macromolecular complexes by blue native gels

Cells were homogenized in 1 ml buffer (83 mM sucrose, 7 mM sodiumphosphate, pH 7.5, 0.3 mM EDTA, 0.7 mM aminocaproic acid) by 30 strokes at 2,000 rpm using a motor-driven tightly fitting 0.5–1 ml glass/Teflon Potter-Elvehjem homogenizer. The homogenate was centrifuged 5 min at 500 g and 4°C. The resulting supernatant was aliquoted according to 20 mg cell wet weight and centrifuged 10 min at 22,000 xg. The pellet containing enriched mitochondrial membrane fraction were resuspended in 40 μl buffer A (50 mM NaCl, 50 mM imidazole pH 7, 1 mM EDTA, 2 mM aminocaproic acid). Membrane protein complexes were solubilized with 10 μl 20% digitonin (w/v in water) to obtain a detergent/protein ratio of 5 g/g. Samples were centrifuged for 10 min at 22,000 xg. 200 μg total protein were supplemented with 2.5 μl 5% Coomassie and 5 μl 50% glycerol and loaded equally onto two 3 to 18% gradient gels. For complexome profiling one BN-gel was stained with Coomassie and was scanned by an office scanner (Epson perfection 2400 PHOTO) for documentation. For identification, second BN-gel was blotted on PVDF membrane and decorated with antibodies against mitochondrial complexes. Chemiluminescence from blots was detected by ChemiDoc XRS system operated by Quantity One Software (Bio-Rad).

### Sample preparation for complexome profiling

Coomassie stained gels were extensively washed with water. Each lane was cut into 60 equal slices and collected in 96 filter well plates (30–40μm PP/PE, Pall Corporation). The gel pieces were destained in 60% Methanol, 50 mM ammoniumbicarbonate (ABC). Solutions were removed by centrifugation for 2 min at 1500 rpm. Proteins were reduced in 10 mM DTT, 50 mM ABC for 1 hour at 56°C and alkylated for 45 min in 30 mM iodoacetamid. Samples were digested for 16 hours with trypsin (sequencing grade, Promega) at 37°C in 50 mM ABC, 0.01% Protease Max (Promega) and 1 mM CaCl_2_. Peptides were eluted in 30% acetonitrile and 3% formic acid, centrifuged into a fresh 96 well plate, dried in speed vac and resolved in 1% acetonitrile and 0.5% formic acid.

### Mass spectrometry

Liquid chromatography / mass spectrometry (LC/MS) was performed on Thermo Scientific^™^ Q Exactive mass spectrometer with an ultra-high performance liquid chromatography unit (Thermo Scientific Dionex Ultimate 3000) via a Nanospray Flex Ion-Source (Thermo Scientific) at the front end. Peptides were loaded on a C18 reversed-phase precolumn (Thermo Scientific) followed by separation on in-house packed 2.4 μm Reprosil C18 resin (Dr. Maisch GmbH) picotip emitter tip (diameter 100 μm, 15 cm long, New Objectives) using a gradient from 5% acetonitrile, 0.1% formic acid to 50% acetonitrile, 0.1% formic acid for 30 min with a flow rate of 400 nl/min. Each run was finished by washout with 80% acetonitrile, 0.1% formic acid and column equilibration in 5% acetonitrile, 0.1% formic acid. Mass spectrometry (MS) data were recorded by data dependent Top10 acquisition selecting the ten most abundant precursor ions in positive mode for fragmentation using dynamic exclusion of 30s. Full MS scan range was 300 to 2000 m/z with a resolution of 70000, and an automatic gain control (AGC) value of 3*10^6^ total ion counts with a maximal ion injection time of 250 ms. Only higher charged ions (2+) were selected for MS/MS scans with a resolution of 17500, an isolation window of 2 m/z and an automatic gain control value set to 5*10^4^ ions with a maximal ion injection time of 150 ms. Lock mass option for 445.120025 m/z (Olsen et al., 2005) and 371.10124 m/z was enabled to ensure high mass accuracy during many following runs.

### Complexome profiling data analysis

X calibur Raw files were analysed by proteomics software Max Quant (1.5.2.8) [45]. The enzyme specificity was set to Trypsin, missed cleavages were limited to 2. Following variable modifications were selected: at N-terminus acetylation (+42.01), oxidation of methionine (+15.99), as fixed modification Carbamidomethylation (+57.02), on cysteines. Human reference proteome set from Uniprot (Download April 2015, 68506 entries) was used to identify peptides and proteins. False discovery rate (FDR) was set to 1%. MaxQuant output file includes peptide and protein identification, accession numbers, protein and gene names, sequence coverage of each sample or gel slice, posterior error probability (PEP) values and intensity-based absolute quantification (IBAQ) values for complexome profiling was prepared. Identifications from reverse decoy database, identified by site and known contaminants were excluded.

Abundance profiles were generated by NOVA software [46] using intensity-based absolute quantification (IBAQ) values from MaxQuant [45]. IBAQ values of proteins from gel lane fraction were normalized to maximum of the lane and hierarchical clustered using Pearson Correlation distance function and average linkage and displayed as heatmaps. For native mass calibration the slice number of the maximum appearance of mitochondrial complex II (123055 kDa), complex III dimer (483695 Da), Complex IV (210786 Da), complex V (618824Da) and respiratory supercomplex containing complex I, III dimer and one copy of complex IV (1654457Da) was used. The equation [f(x) = 27240*e^(0.0936x), R^2^ = 0.9918] obtained by exponential regression was used to calculate the native masses of each slice.

## Supporting Information

S1 Fig Mitochondrial area and cristae density is more in MIC13 KO cells. (A) Box blot indicating the total mitochondrial area in control and MIC13 KO cells (*, p value < 0.05; **, p value < 0.01; ^#^, p value = n.s.). (B) Box blot showing cristae area (per mitochondria) in control and MIC13 KO3 (****, p value < 0.0001).
(TIF)

S2 Fig Complexome analysis of respiratory chain complexes in control and MIC13 KO1 cells. (TIF)

S3 Fig Mitochondrial morphology of MIC13 KO cells. Mitochondrial morphology of control and MIC13 KO cells analyzed by cytochrome *c* staining. The lower panel shows the zoomed image of the box in upper panel. Scale bar 10μm.
(TIF)
