# Supplementary figures and images for: Mic13 Is Essential for Formation of Crista Junctions in Mammalian Cells
Source: PLoS One. 2016 Aug 1;11(8):e0160258. doi: 10.1371/journal.pone.0160258 (PMC4968808; doi:10.1371/journal.pone.0160258)

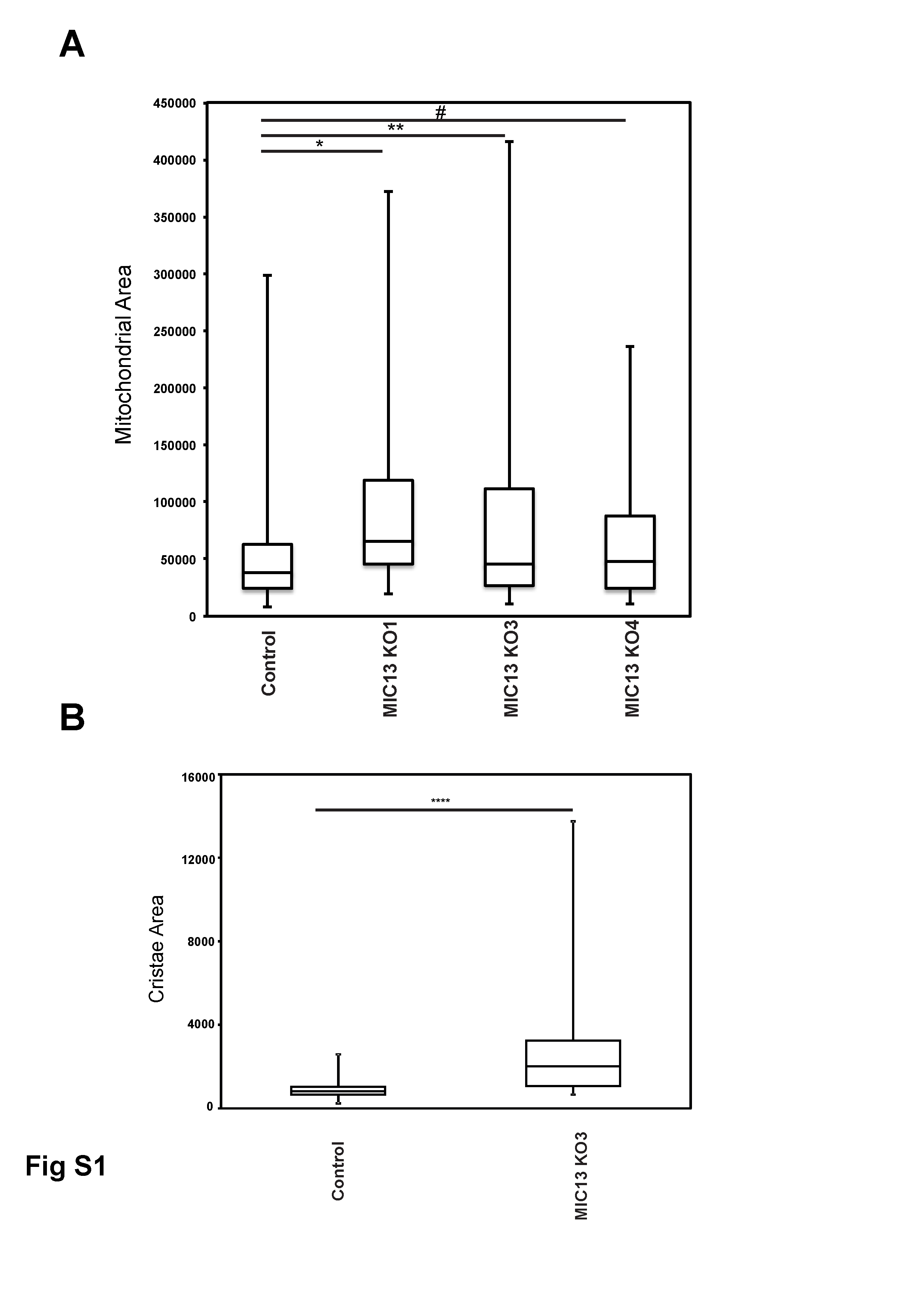

Supplement: S1 Fig — (A) Box blot indicating the total mitochondrial area in control and MIC13 KO cells (*, p value < 0.05; **, p value < 0.01; #, p value = n.s.). (B) Box blot showing cristae area (per mitochondria) in control and MIC13 KO3 (****, p value < 0.0001). (TIF) [file pone.0160258.s001.tif]

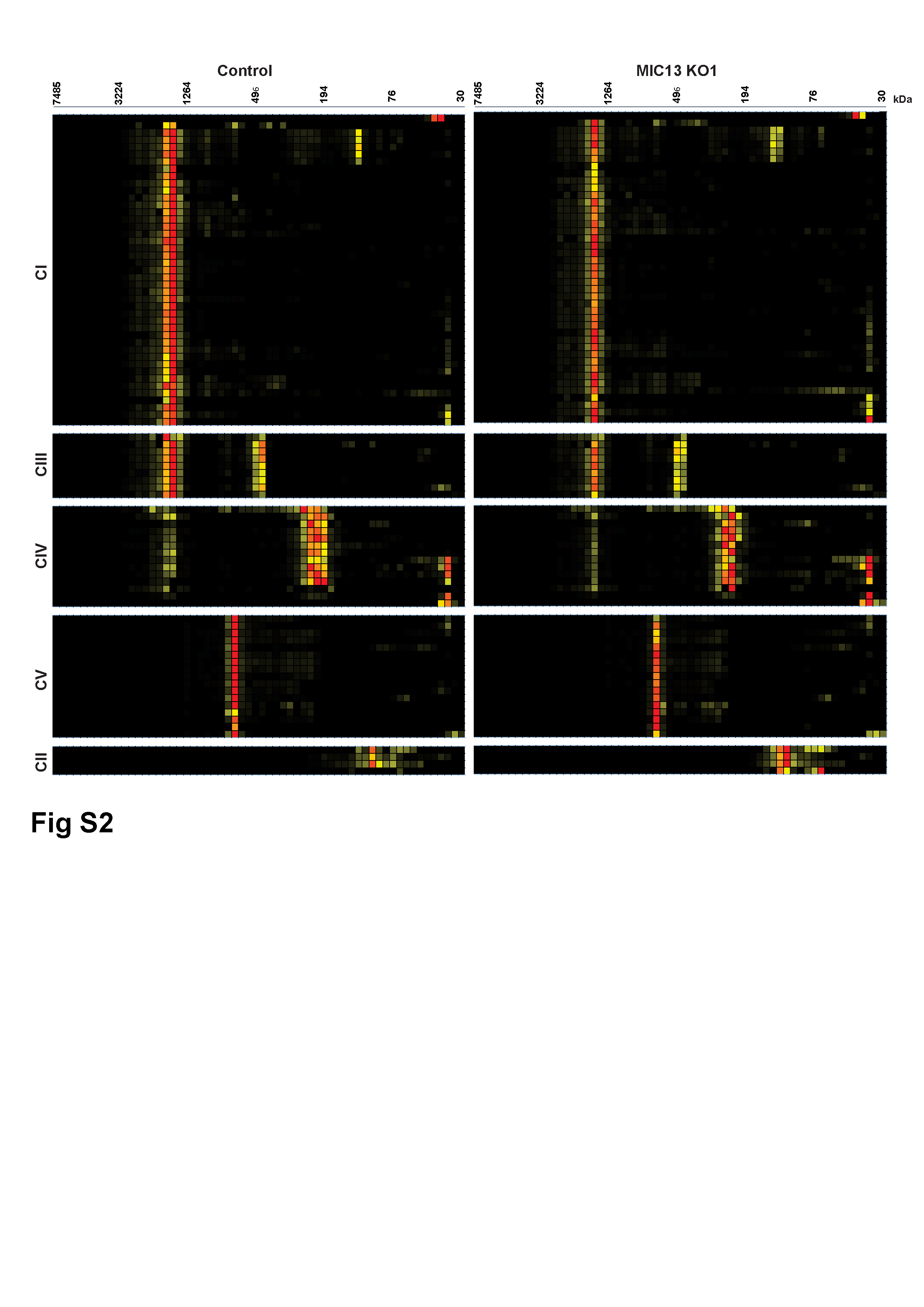

Supplement: S2 Fig — (TIF) [file pone.0160258.s002.tif]

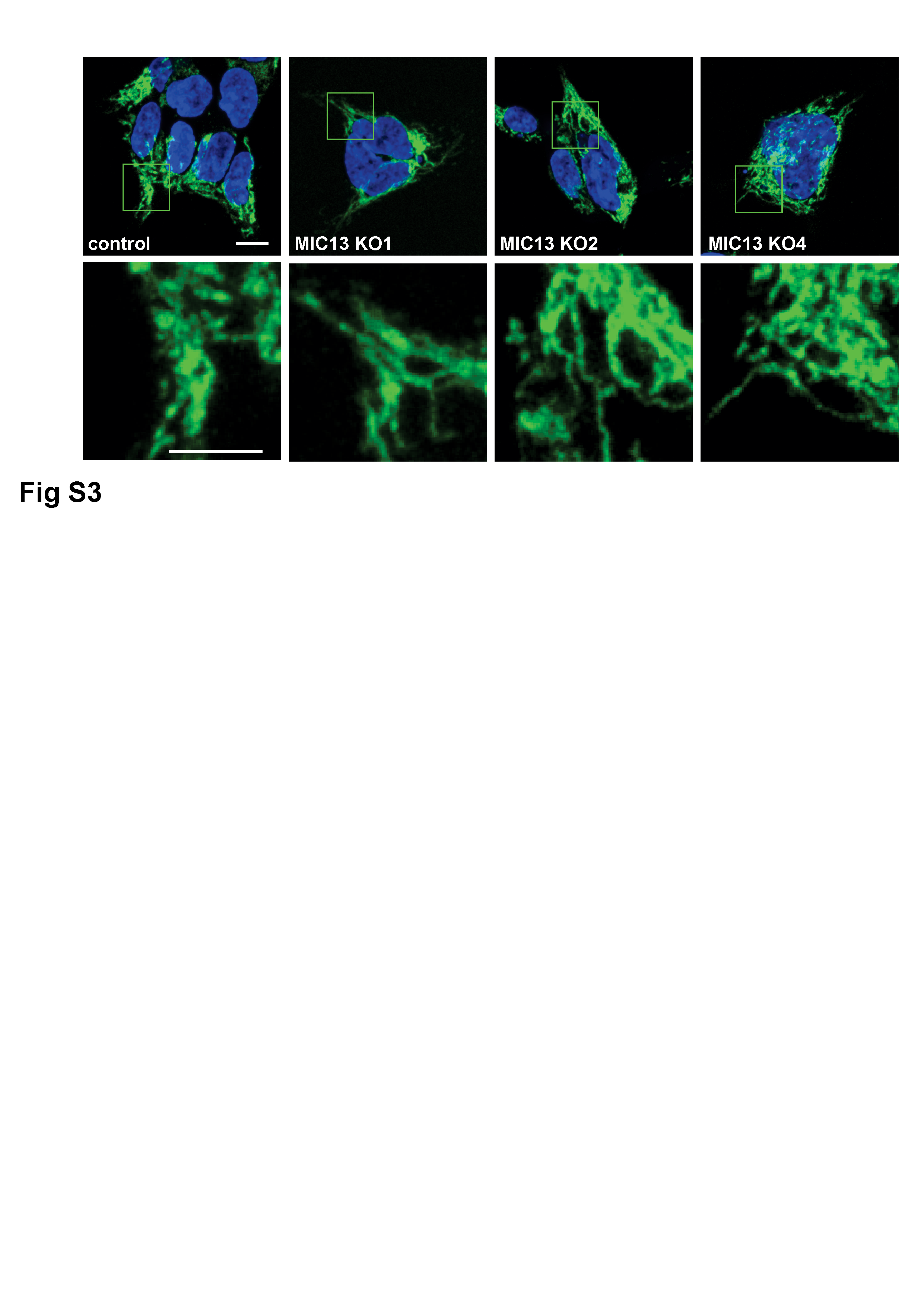

Supplement: S3 Fig — Mitochondrial morphology of control and MIC13 KO cells analyzed by cytochrome c staining. The lower panel shows the zoomed image of the box in upper panel. Scale bar 10μm. (TIF) [file pone.0160258.s003.tif]
